# Novel Light Convolutional Neural Network for COVID Detection with Watershed Based Region Growing Segmentation

**DOI:** 10.3390/jimaging9020042

**Published:** 2023-02-13

**Authors:** Hassan Ali Khan, Xueqing Gong, Fenglin Bi, Rashid Ali

**Affiliations:** 1 Software Engineering Insitute, East China Normal University, Shanghai 200062, China; 2 School of Data Science and Engineering, East China Normal University, Shanghai 200062, China; 3 School of Information Engineering, Southwest University of Science and Technology, Mianyang 621010, China; 4 Department of Computer Science, University of Turbat, Turbat 92600, Pakistan

**Keywords:** convolutional neural network, COVID-19, classification, CNN, segmentation, watershed segmentation, X-rays, CT scans

## Abstract

A rapidly spreading epidemic, COVID-19 had a serious effect on millions and took many lives. Therefore, for individuals with COVID-19, early discovery is essential for halting the infection’s progress. To quickly and accurately diagnose COVID-19, imaging modalities, including computed tomography (CT) scans and chest X-ray radiographs, are frequently employed. The potential of artificial intelligence (AI) approaches further explored the creation of automated and precise COVID-19 detection systems. Scientists widely use deep learning techniques to identify coronavirus infection in lung imaging. In our paper, we developed a novel light CNN model architecture with watershed-based region-growing segmentation on Chest X-rays. Both CT scans and X-ray radiographs were employed along with 5-fold cross-validation. Compared to earlier state-of-the-art models, our model is lighter and outperformed the previous methods by achieving a mean accuracy of 98.8% on X-ray images and 98.6% on CT scans, predicting the rate of 0.99% and 0.97% for PPV (Positive predicted Value) and NPV (Negative predicted Value) rate of 0.98% and 0.99%, respectively.

## 1. Introduction

Coronaviruses represent a diverse potential source of infection. Coronaviruses acquires their name from the spike proteins that emerge from them, giving them a crown-like appearance. These spike proteins are crucial to the viruses’ metabolism. The spike protein is the portion of the virus that infects a human cell and allows it to multiply and spread to other cells. The SARS-CoV-2 virus, which causes COVID-19, was identified in Wuhan, China, in December 2019. Shortness of breath, muscle aches, fever, sore throat, and some severe disorders, including Middle East respiratory syndrome (MERS) and severe acute respiratory syndrome (SARS) caused by COVID-19 [1]. Symptoms appear in 2 to 14 days after inoculation of the virus. A person infected with the coronavirus can be dangerous to others for up to two days before clinical signs appear and for 10 to 20 days after that, depending on the patient immune system and the seriousness of their infection. COVID-19 is a contagious disease; therefore, its mutation may allow the coronavirus to transmit more quickly from person to person, as seen with the delta and omicron variations.

According to the World Health Organization, the overall number of cases has already surpassed 516,900,683, and the total number of deaths has exceeded 6,259,945. Regarding reported cases worldwide, Europe and America were top of the list [2]. The medical community faced additional hurdles as a result of this infection. Infected persons had no access to medical wards or ventilators, and even a scarcity of physicians and nurses in hospitals. Various governments have introduced new policies and adopted new ways of living. Many specialists and scientists worked on making vaccines to prevent the virus; however, the vaccines slow the virus’s spread and make it easier to develop immunity by forming specific antibodies. A vaccine was just a prevention strategy, not a cure. Therefore, numerous challenges were encountered with vaccine administration, especially vaccine availability. The RT-PCR of respiratory tract samples was the most prevalent and standard approach for diagnosing COVID-19. RT-PCR test kits were often used to identify COVID-19. However, the RT-PCR test’s complexity, low sensitivity, and time consumption caused a lot of concern. The shortage of test kit availability can create a crucial problem. Additionally, it will become quite difficult and expensive to run these tests frequently and regularly for the population as the number of infected individuals grows. As a result, specialists have switched to chest CT scans and X-rays to detect COVID-19, which is faster and more accurate than the RT-PCR test. However, using these CT scans or X-ray imaging techniques can sometimes be challenging since experts cannot always make a distinction between COVID-19 and other respiratory infections such as seasonal flu [3].

Processing medical images have become increasingly popular. Deeper and more accurate network models that can compete with humans in terms of speed and accuracy have recently received much interest and increased medical research considerably. Researchers have been working with doctors and clinical professionals to detect coronavirus infections early. Computer-assisted diagnostic methods that can assist radiologists in immediately and precisely interpreting radiography images are very popular. Deep learning models have proven to be effective for image classification and detection. Medical imaging has recently gained much attention due to the rise of deep-learning approaches for computer-aided analysis of respiratory illnesses. Creating automated systems for COVID-19 detection is only possible using Chest X-rays or CT scan images. For the early diagnosis of coronavirus infection from images such as CT scans or X-ray images, many researchers have already applied a variety of deep learning and machine learning approaches to detect the virus. However, any automated system that intends to be used in practice must have a high detection rate and reliable performance on testing datasets. Data that are multimodal and of acceptable performance can yield it to trial. Therefore, in our research, we used a Novel light Convolutional Neural Network model along with a Region-based Watershed Image segmentation technique to classify COVID-19, Pneumonia, and Normal images. We conducted extensive research and used CT scans and X-ray images to enhance the effectiveness of COVID-19 detection and more accurately analyze the performance of our proposed model by using 5-fold cross-validation.

## 2. Related Work

Since the worldwide spread of COVID-19, the demand for faster and more accurate detection through X-ray and CT image interpretations has prompted several researchers to develop artificial intelligence-based systems that ensemble deep learning and their performance were measured based on accuracy, sensitivity, and specificity. Many research papers have addressed the use of machine learning and convolutional neural networks in detecting diseases based on medical images, with impressive results. A few of the earlier state-of-the-art studies that used Deep learning methods to diagnose COVID-19 images are Muhammet Fatih [4] employed five different pre-trained CNN models to extract features from COVID patient’s chest X-ray images, along with ANN segmentation and hypermeter determination using Machine learning methods for Bayesian optimization. The DenseNet201 model with the SVM method Bayesian optimization obtained the highest accuracy of 96.29%. Gour Mahesh [5] developed UA-ConvNet, an uncertainty-aware deep neural network CNN model using CXR images, an automatic COVID-19 disease diagnosis. Three distinct chest X-ray image datasets have been utilized to assess the suggested approach. The G-mean of the proposed UA-ConvNet model was 98.02%, with a sensitivity of 98.15%. Rubina Sarki [6] exhibited a scratched CNN architecture that she trained on three datasets and compared to VGG16, InceptionV3, and Xception models using a transfer learning technique. The presented CNN model has a 93.7% accuracy compared to 87.5% for the transfer learning model. Mohamed Loey [7] applied a novel Bayesian optimization-based CNN model to extract the features from chest X-ray images, and Bayesian-based optimization was used to tune the CNN model hyperparameters. The proposed method had 96% accuracy. Pedro R. A.S Bassi [8] used a nonlinear transfer learning strategy to generate heatmaps from a chest X-ray dataset using Layer-wise Relevance Propagation and employing DenseNet201 and ChexNet pre-trained models. On the test dataset, the proposed approach achieved 100% accuracy. Nurul Absar [9] employed CXR images for data augmentation and used a transfer learning approach utilizing the SqueezeNet model for extracting features. For classification, the Support Vector Machine Algorithm was used. On CXR images, the experiment achieved 98.8% accuracy. Muhammad Aftab [10] developed a deep learning model to distinguish between normal and infected X-ray patient images and then used an LSTM network model to further classify the infected X-ray images into influenza and COVID-19 patient images. Muralidharan Nehat [11] applied a Fixed Boundary Range based Two-Dimensional EWT filter on X-ray images, and the images were decomposed into multiple modes. Using a multiscale deep Convolutional Neural Network, these evaluated mode images were classified into Normal, Pneumonia, and COVID-19 instances. The suggested method obtained an accuracy of 96% and 97.17% on two different datasets.

Some researchers used CT scan images to identify COVID-19 patients. CT scan is a more advanced technique for evaluating the severity of infection in various parts of the chest because CT scan offers 3D imaging of organs from various angles. Md. Robiul Islam [12] employed Contrast Limiting Histogram Equalization for preprocessing phase over CT images to improve image pixel density and developed a new Convolutional Neural Network (CNN) model to extract features from 2482 CT scan images. The proposed scheme succeeded, by achieving 99.73% accuracy, 99.46% precision, and 100% recalls. On CT scan images, S. V. Kogilavani [13] utilized a transfer learning approach and fine-tuned pre-trained models, including VGG-16, MobileNet, DeseNet121, EfficientNet, Xception, and NASNet. The VGG-16 model seemed to have the highest accuracy, with a score of 97.68%. Mei-Ling Huang [14] suggested LightEfficientNetV2 as a lightweight CNN model for detecting COVID-19, Pneumonia, and Normal employing X-ray and CT images and compared its performance with pre-trained models such as InceptionV3, Xception, ResNet50, MobileNetV2, DenseNet121, EfficientNetV2, and EfficientNet-B0. On different datasets, the LightEfficientNetV2 model proposed in this study achieved 98.33% and 96.78% accuracy, respectively. The Sparrow search algorithm (SSA) was introduced by Nadiah A. Baghdadi [15] to optimize the CNN and transfer learning model parameters to select the optimal configuration. The pre-trained models employed in the proposed study were MobileNet, MobileNetV2, SeresNext50, SeresNext101, SeNet154, MobileNetV3Small, and MobileNetV3Large. With the MobileNetV3Large model, the suggested Framework achieves the highest accuracy of 99.74%. Hasija Sanskar [16] performed multiclassification on CT images using various CNN models, then binary classification on COVID-positive and COVID-negative CT images in the first phase, and then COVID negative images were categorized into Pneumonia positive and Pneumonia negative in the second phase. In comparison to existing pre-trained models, the proposed method obtained 98.38 percent accuracy. Murat Canayaz [17] implemented a strategy based on Bayesian Optimization (BO) with pre-trained MobilNetv2 and ResNet-50 models, along with KNN and SVM machine learning algorithms. With the KNN algorithm, the BO parameters had the highest accuracy of 99.37%.

In previous research, researchers applied various deep learning and machine learning approaches to detect the virus. Most studies employed transfer learning approaches to detect COVID-19 patients, and only a few suggest a novel CNN architecture that was competitive to transfer learning-based approaches in terms of performance. However, they also encountered challenges with limited accuracy in the analysis and testing of the proposed solutions. X-ray images sometimes develop noise from the radiation, resulting in more intense gray pixels on an image. Only a few researchers applied image processing techniques for scaling and normalization, while the majority of researchers used noisy datasets. Therefore, dealing with that kind of noisy dataset is also essential to obtain a clear image of the lungs. In previous research, smaller datasets were used, and a data augmentation strategy was applied to target either X-ray or CT images. While in our work, we used both X-ray images and CT scans dataset and applied a watershed-based region-growing segmentation to segment the region of interest, removed the noise in our X-ray images, and used a sequential Novel light CNN architecture to extract features from both X-ray and CT scans. Due to its light architecture consisting of just seven convolution layers and two fully connected layers, the model works efficiently and needs less computational time. For optimization, we used Adam optimizer to reduce loss. We used 5-fold cross-validations on our dataset to evaluate the model robustness, while in previous work, a very small number of studies used cross-validation. Moreover, as mentioned in the related work, we contrasted our model’s performance with state-of-the-art models.

## 3. Materials and Method

**Figure 1:**
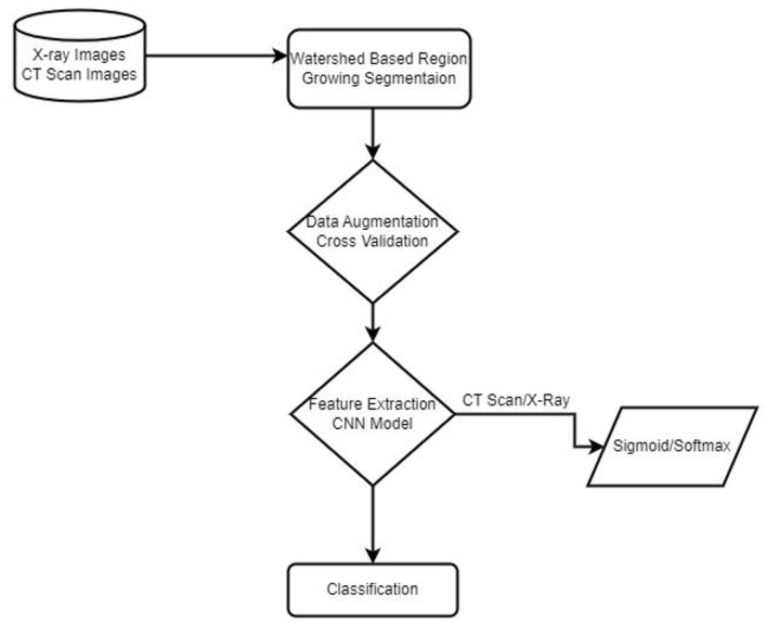
Proposed Methodology.

In the proposed method, we used two different datasets and first applied image processing techniques to remove noise and segment the region of interest in COVID-19 X-ray images. Then we applied data augmentation and proposed a Sequential novel light CNN model for classification with 5-fold cross-validation on our dataset to obtain an optimal model. We compared our model accuracy with the previous state of the Arts. An overview of the proposed model is shown in Figure 1.

### 3.1. Dataset

**Figure 2:**
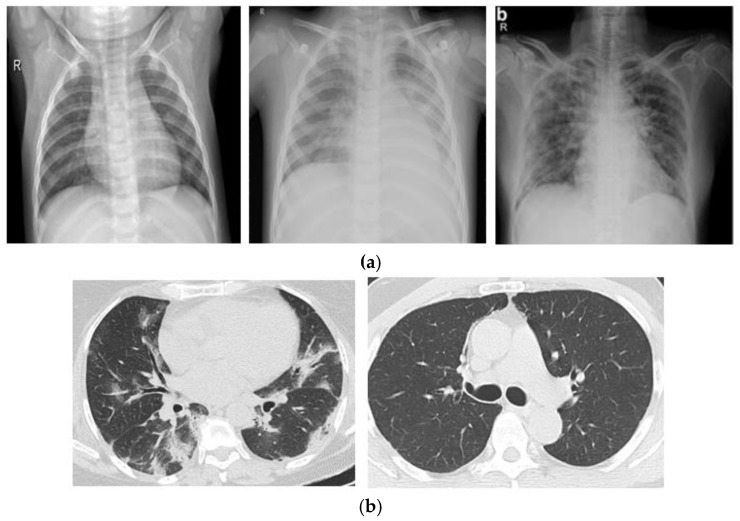
(**a**) X-ray Radiographs of normal, pneumonia and COVID patients. (**b**) CT Scan images of COVID and normal patients.

In our study, we conducted experiments utilizing two different kinds of image datasets. One is X-ray radiographs of the chest, which have extensively been employed in previous research. The X-ray radiograph dataset consists of three classes: normal, pneumonia, and COVID patient chest X-rays. The COVID-19 radiography database was developed by a team of medical experts and researchers worldwide, and it is publicly available on Kaggle [18]. The dataset contains 3829 X-rays images of Normal, Pneumonia, and COVID-19 patients. The second dataset consists of CT scan images containing 2482 CT scans of Normal and COVID patients. The data were obtained from patients diagnosed at hospitals in Sao Paulo, Brazil, and are openly accessible for study and research development [19]. The most common diagnostic imaging test is the X-ray, which is easily accessible. CT scans are more time-consuming than X-rays but are quick and precise. Therefore, we only used the image processing technique on X-ray images in our dataset. The dataset from the CT scan images had already been processed. Two separate datasets are displayed in Figure 2.

### 3.2. Watershed Based Region Growing Segmentation

Raw images were used as input in previous case studies; however, it may not be better to apply classification methods directly to original datasets because the accuracy result is altered, which causes misclassification. Other issues encompass image imbalance difficulties and the fact that patients’ chest X-ray images are not often aligned. The position of the lungs varies from one image to another. Using X-ray images, the chest has to be the region of interest for COVID-19 diagnosis. As a result, the segmentation method has been used to segregate the pixels of interest and detect the active contours in X-ray images, enabling the classifier to produce more accurate predictions. The efficacy of conducting lung segmentation by applying CNN on CXR images to identify COVID-19 is evaluated by Lucas O. Teixeira et al. [20]. However, as we can see in the mentioned literature, the segmentation was done using U-Net and CNN. That requires a lot of computational power, Moreover, for classification purposes, they used a transfer learning approach and used pre-trained models such as VGG, RestNet, and Inception. However, we employed a watershed-based region-growing segmentation method, which grows regions nonlinearly by integrating neighbor pixels that are similar and associated to the seed pixel and removing pixels with irregularly shaped watersheds. When no neighbor pixels fulfill the similarity condition, the growth ends. The watershed algorithm considers the values of pixels to be a local topography (elevation). The method floods basins from the markers until watershed lines connecting various basins meet.

**Figure 3:**
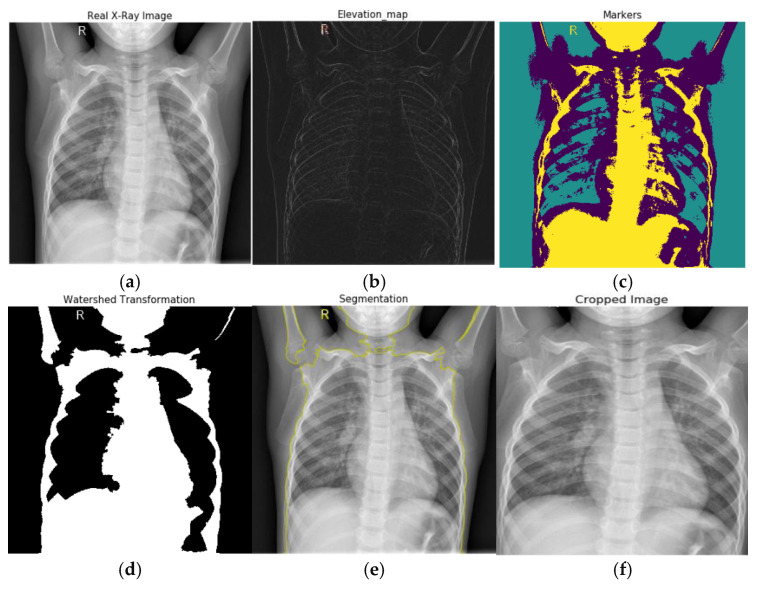
Watershed based region growing segmentation: (**a**) real X-ray image, (**b**) Sobel edge detection and elevation map, (**c**) setting markers, (**d**) watershed transformation, (**e**) plotting overlays, for segmentation, and (**f**) image cropping.

In our experiment, first, we converted our X-ray images into grayscale. Then we used a Sobel edge detection filter to compute the estimated absolute gradient at each region in the grayscale image. After applying Sobel edge detection to emphasize edges on our images, we employed an elevation map to display the various altitudes in a region. Contour lines connect points on a map showing areas with similar elevation levels and are used to illustrate elevation on maps. After that, we set our markers on these X-ray images. Then, we employed a morphological watershed transformation technique to segment watershed regions. The watershed can run and identify the exact boundaries due to our defined markers. We fill the regions separated from the background by thin patches during the watershed. Finally, we plotted overlays and contours around the processed image and cropped the segmented chest images from the X-ray images. The segmentation process is displayed in Figure 3. The real X-ray image, as seen in Figure 3, has many black pixels around the borders, and there is some texture information on the X-ray image; if we feed this image straight to our CNN model, it will extract these dark edges portions as well. As a result, we segmented the area of interest in the X-ray images and provided only the region of interest as an input to our neural network model.

### 3.3. Data Augmentation

**Figure 4:**
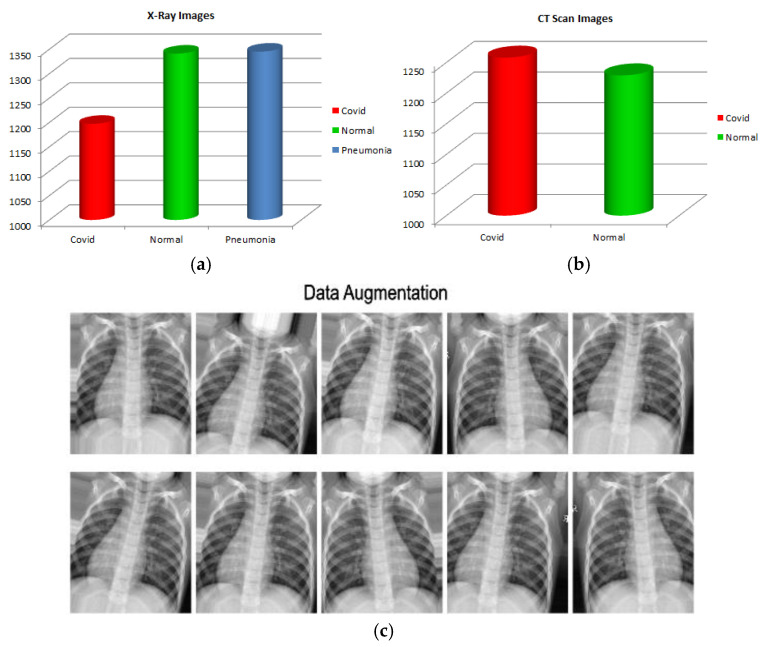
(**a**) X-ray images class imbalance, (**b**) CT scan class imbalance, and (**c**) data augmentation.

In image processing or computer vision, data imbalance is a major problem, particularly in the medical field. Data augmentation helps to enhance the number of samples and provide diversity in the dataset without collecting new samples. It rectifies the class imbalance by producing synthetic examples of classes with fewer objects. The input images for every batch were normalized during data augmentation. On each image, translation and rotation were applied. To improve the variety of data available, rotation creates the same image at several angles, and in translation, images are moved across the X or Y axes. These procedures alter the image’s shape and pixel values so that the human visual system can observe it easily. Thus, the data augmentation feature offers more effective training as more diverse images were used in the deep neural network’s training. Therefore, we used the data augmentation approach on both CT scans and segmented X-ray images by flipping and rotating the images between 0 and 360 angles, along with images normalization. Figure 4 displays the data’s class imbalance and multiple augmented images created from a single image.

### 3.4. Convolutional Neural Network

**Figure 5:**
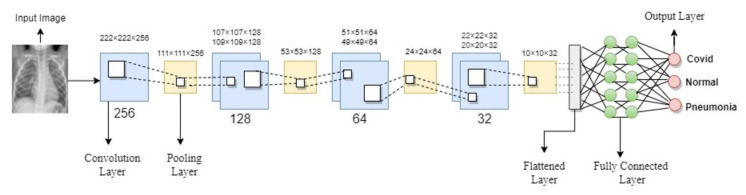
Proposed CNN Model Architecture.

A CNN deep learning model comprises many convolutional layers that extract detailed and discrete features from the input images, a pooling layer to reduce the network’s parameters and computations, and fully connected layers that add weight matrices and bias vectors to the input to help classify the data. Sometimes, rather than developing a new CNN framework from scratch, an alternatively pre-trained CNN model was used that has already been proved in terms of accuracy and performance. These pre-trained models were employed using a transfer learning approach. Most approaches employed in the prior literature for COVID-19 detection had utilized transfer learning techniques. However, in our study, we put forward a novel light convolutional neural network model to detect COVID-19 cases in both X-ray and CT scan images. We developed a novel light CNN model to extract features from X-ray and CT images having 224 × 224 input sizes. Figure 5 depicts the architecture of the model we proposed.

Our proposed model has only seven convolution layers, making it more efficient and requiring less computational power during the training phase. The receptive field is as small as it can be to obtain the feature map. In our model, every hidden layer has a Rectifier linear unit (ReLU) activation function that can produce a real zero value for negative inputs. ReLU avoids the issue of vanishing gradients due to its linear behavior, which makes gradients proportional to node activation. In the first hidden layer, we added a single convolution layer of 256 filters with a rectifier linear unit (ReLU) function with a pooling layer on top of the convolutional layer to calculate the maximum pixels to sum the patches of the convolution layer. Then we reduced the convolutional layers filters to 128, 64, and 32. We added two fully connected layers with a dropout layer of 0.5 in addition to the convolutional and pooling layers that were followed by an output layer with a softmax and sigmoid function. Since both binary and categorical classification are involved, sigmoid is utilized for binary classification and softmax for categorical classification.

(1) $$Sigmoid\;\left(x\right)=\frac{1}{1+e^{-x}}$$

(2) $$Softmax\;\left(Z_{j}\right)=\frac{e^{z_{i}}}{\sum_{j=1}^{K}e^{z_{j}}}$$

In addition, we employed binary cross entropy for CT scan images and categorical cross entropy for X-ray images to calculate the loss between algorithmically predicted values and the actual label’s value.

(3) $$J\left(z\right)=\;y\;\log\;P\left(y\right)+\left(1-\;y\right)\;\log\left(1-\;P\left(y\right)\right)\;$$

P(y) represents predicted labels, whereas y represents actual labels. Since y is multiplying by log, the whole first term will be zero when the actual labels have a value of 0. Additionally, when y equals 1, the second term will equal zero (1-y), and it will be multiplied by the log.

(4) $$A=-\underset{i=1}{\sum }\;(y_{i}\;log\;P\left(y_{i}\right))$$

(5) $$A=-y_{1}\;log\;P\left(y_{1}\right)-y_{2}\;log\;P\left(y_{2}\right)\ldots -y_{n}\;log\;P\left(y_{2}\right)$$

The labels are one hot encoded in categorical cross-entropy, and only the positive class label will be 1 while the others will be 0. Therefore, because there are 0 target labels when the term is multiplied by log, it will result in 0. The loss function will only retain the positive class term that is 1.

### 3.5. Optimization

After calculating the difference between our actual and predicted values through the loss function, we attempt to minimize the difference by updating the weights and learning rates in an optimization method. Depending on the algorithm used for optimization, weights and learning rates may need to be adjusted to minimize the loss. Therefore, we applied the Adaptive Moment Estimation (Adam) optimizer to reduce the loss in the experiment’s performance. The Adam optimizer is Stochastic Gradient Descent with Momentum and RMS prop combination.

(6) $$W=W-\eta \left(\frac{V_{dW\;}}{\sqrt{S_{dW\;}+\varepsilon \;}}\right)\;$$

(7) $$b=b-\eta \left(\frac{V_{db\;}}{\sqrt{S_{db\;}+\varepsilon \;}}\right)$$

η is the learning rate with a range of values, while *S_dw_* and *S_db_* are the exponentially weighted means of the squares of the previous gradients (RMS Prop) for the corrected weights and bias, respectively; *V_dw_* and *V_db_* are the exponentially weighted means of past gradients (SGD). Epsilon Ɛ inhibits the division of zero.

## 4. 5-Fold Cross Validation

**Figure 6:**
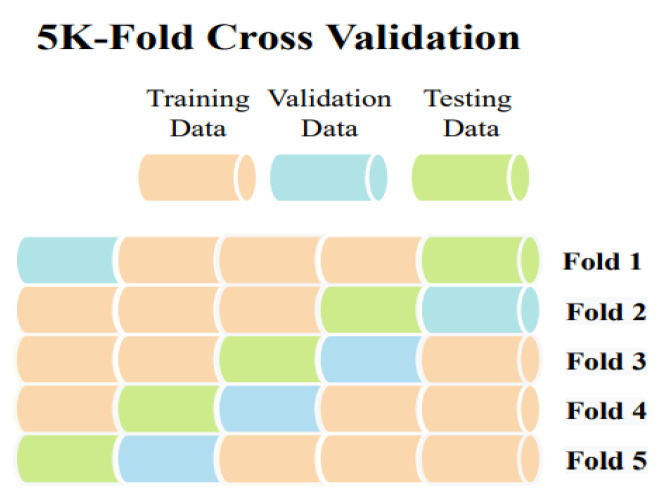
5-Fold Cross Validation.

Sometimes, due to the scarcity of a large dataset, a cross-validation approach is utilized in the medical imaging area to evaluate a model’s robustness and performance. The dataset is partitioned into k sections using k-fold cross-validation. One partition is preserved as validation data for testing the model, while the remaining k-1 partitions are used as training data. The cross-validation process is then repeated k times. Consequently, K-fold cross-validation was used to assess the performance of our model, with a k value of 5. For training data, it will be k-1 so four chunks for training data and one chunk for testing data. The cross-validation procedure was carried out five times. Each time, the dataset was shuffled before creating the data chunks. The 5-fold cross-validation’s core mechanism is shown in Figure 6.

## 5. Results and Discussion

**Figure 7:**
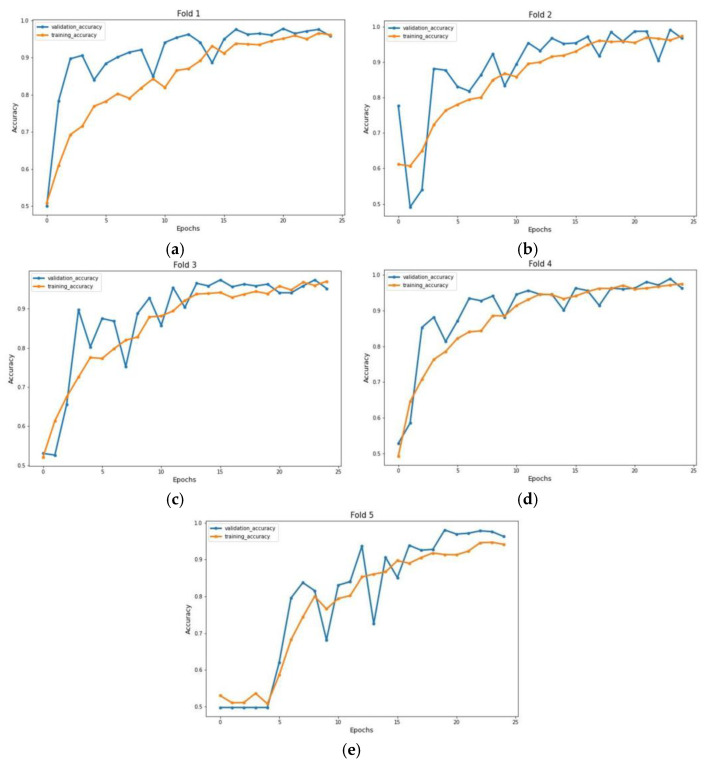
Accuracy graph of CT scan images during training phase: (**a**) Fold 1, (**b**) Fold 2, (**c**) Fold 3, (**d**) Fold 4, and (**e**) Fold 5.

**Figure 8:**
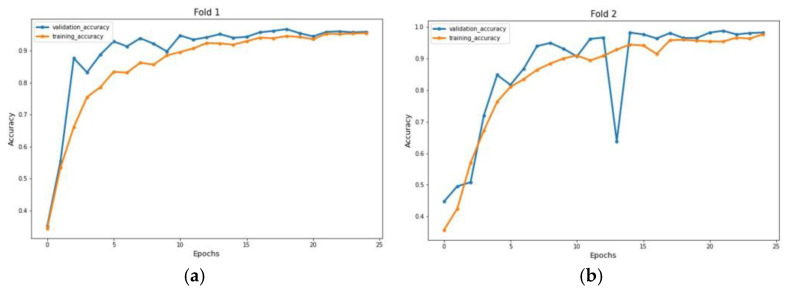

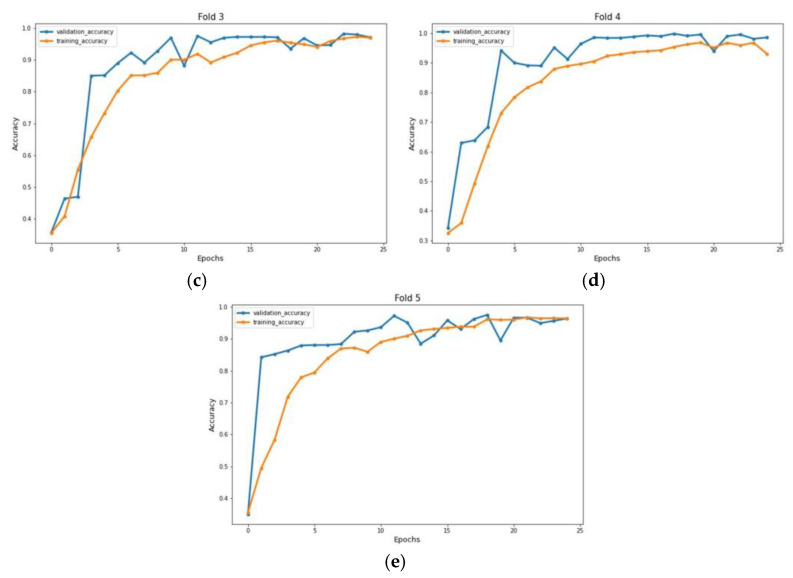
Accuracy graph of X-ray radiographs during training phase: (**a**) Fold 1, (**b**) Fold 2, (**c**) Fold 3, (**d**) Fold 4, and (**e**) Fold 5.

The experiment was carried out on two distinct COVID-19 datasets. The first dataset consisted of CT scans images, and the second was X-ray images. This COVID-19 image dataset was compiled from publicly available websites. We used python language in jupyter notebook and employed the Keras library with Tensorflow 2 as a backend. The NVIDIA RTX 32GB GPU was used for the experiment. We trained the model for 25 epochs having a batch size of 64. Moreover, we applied data augmentation using the Keras Image Data Generator function. Our proposed model used a checkpoint to retain the maximum validation accuracy during the epochs training. The training and testing phases do not encompass modifying the chosen hyper parameters except for the loss and activation function in the final layer. Figure 7 and Figure 8 illustrate the accuracy of training and validation data for CT scans and X-ray images for each fold.

We compute the accuracy, positively predicted value, and negative predicted value on each testing datum from each fold of our dataset to analyze the performance of our proposed model. Here, the ratio of patients diagnosed with positive COVID to all who had positive COVID test results is the positive predicted value. In contrast, the negative predicted value shows the fraction of people who have received a true negative diagnosis for COVID compared to all negative test results of COVID. Table 1 shows the accuracy, positive predicted value (PPV), and negative predicted value (NPV). PPV and NPV are calculated as

(8) $$Positive\;Predicted\;Value=\frac{True\;Positive}{True\;Positive+False\;Positive}$$

(9) $$Negative\;Predicted\;Value=\frac{True\;Negative}{True\;Negative+False\;Negative}$$

(10) $$Accuracy=\;\frac{True\;Positive+True\;Negative}{Total\;No\;of\;Predictions}\times 100$$

**Figure 9:**
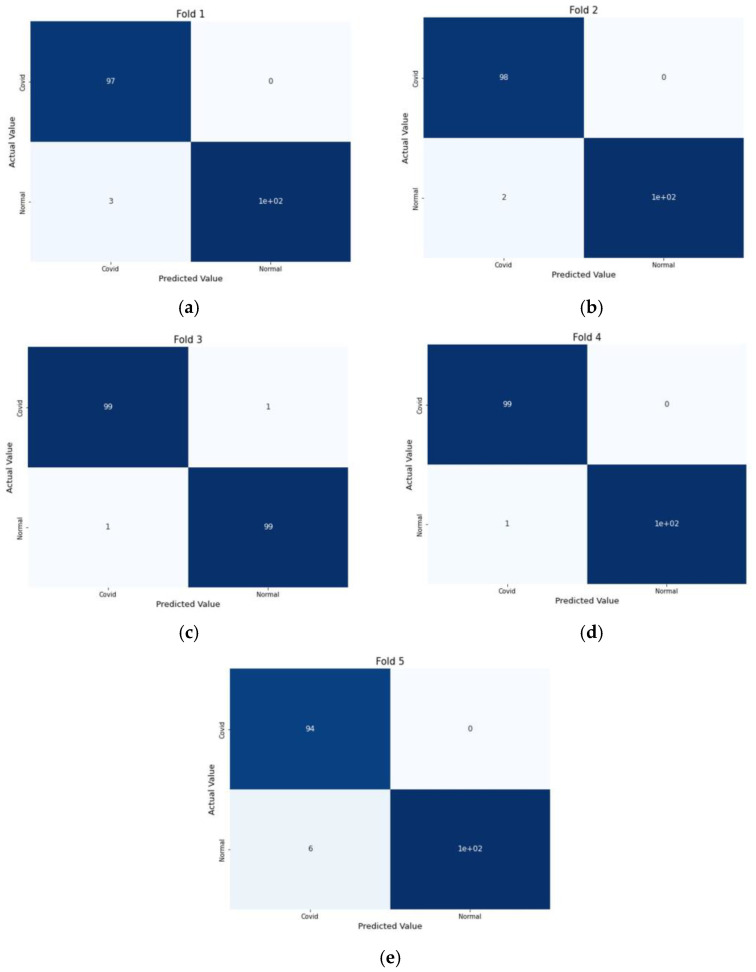
Confusion Metrics for CT scans on 5-fold: (**a**) Fold 1, (**b**) Fold 2, (**c**) Fold 3, (**d**) Fold 4, and (**e**) Fold 5.

**Figure 10:**
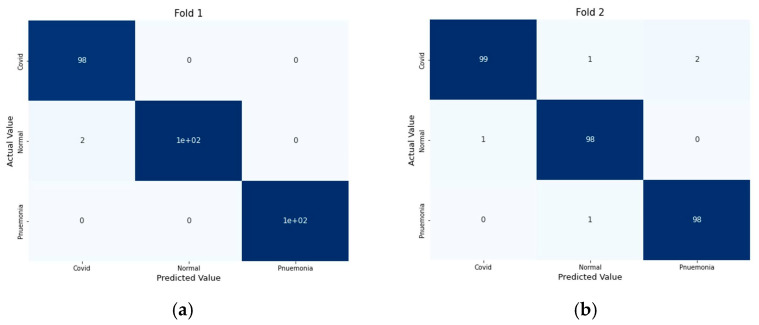

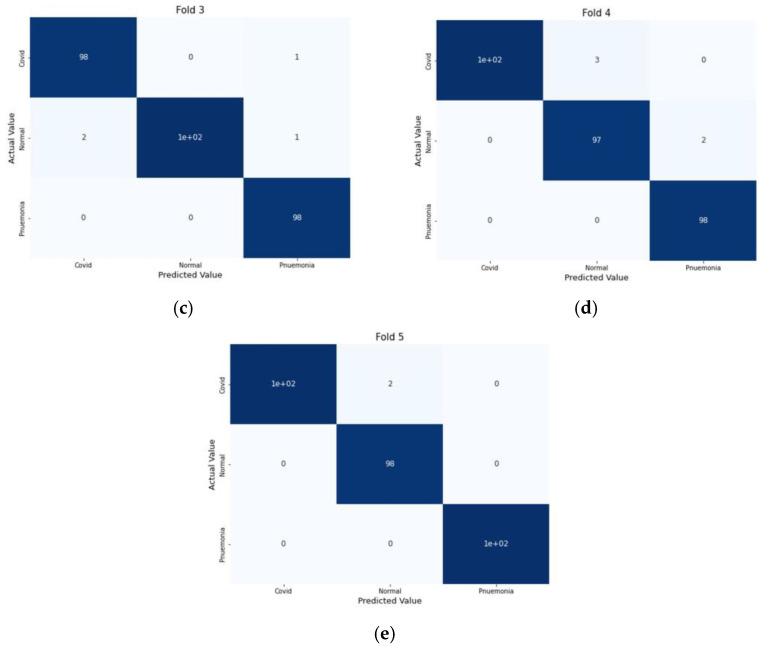
Confusion Metrics for X-ray images on 5-fold: (**a**) Fold 1, (**b**) Fold 2, (**c**) Fold 3, (**d**) Fold 4, and (**e**) Fold 5.

For performance evaluation, the confusion matrix was additionally presented for each fold both for CT scans and X-ray images Figure 9 and Figure 10.

## 6. Performance Comparison

Table 2 compares the accuracy level between our research and the previous state of the arts. Considering this, the suggested approach is analogous to earlier state-of-the-art research. Compared to prior studies, this study is successful due to two key factors. The lungs portion, the region of interest, was first separated from the original images and given to the proposed CNN model. The second is that, as we are familiar with, CT scan images are processed differently from X-ray images. Thus, we employed two unique datasets of CT scan and X-ray images and proposed a novel light CNN model for extraction. Additionally, we used 5-fold cross-validation on our dataset to assess the stability of our CNN model. It can be seen from Table 2 that the proposed approach significantly outperforms the existing methods with a 98.8% and 98.6% mean accuracy rate.

## 7. Conclusions

Prevention of the COVID-19 pandemic depends on early diagnosis. For early detection, different states of the arts were proposed by researchers using deep learning models. This work proposed a novel light method for detecting COVID-19 patients from X-ray and CT scan images. One of the most critical elements influencing the accuracy is the segmentation of lung areas in X-ray images. Therefore, we employed watershed-based region-growing segmentation to identify the region of interest in our images. Second, for the feature extraction, we proposed a novel light convolutional neural network model. Comparing our light neural network architecture to the previous state of arts, it requires lower computation power as our model is very light, having only seven convolutional layers, and it is certainly applicable in a realistic scenario, allowing it to perform multimodal COVID-19 detection employing both X-ray and CT scan images. Attributes from multiple datasets will probably have varying sizes and meanings. The CNN architecture should be adaptable to the kind of data we feed to our CNN model to deliver the interpretation and meaning from different datasets. We employ two types of datasets for categorical and binary classification. In order to effectively identify COVID-19, our proposed methodology is quite useful.

Moreover, relatively few publications in the literature employed cross-validation on a dataset, whereas we used 5-fold cross-validations on our dataset. We contrasted the performance of our proposed approach with the most recent ones mentioned in the section related to work. Our model’s performance resulted in the highest accuracy among the models being studied.

## Figures and Tables

**Table 1:** Model classification performance.

Dataset	Fold	PPV	NPV	Accuracy
CT Scan	1	0.97	1.0	98.5
	2	0.98	1.0	99
	3	0.99	0.99	99
	4	0.99	1.0	99.5
	5	0.94	1.0	97
X-ray Images	1	0.98	1.0	99.3
	2	0.99	0.98	98.3
	3	0.98	0.99	98.6
	4	1.0	0.97	98.3
	5	1.0	0.99	99.3

**Table 2:** Performance Comparison with previous state of arts.

Previous Studies	Image Type	Methodologies	Accuracy
Muhammet Fatih et al. [4]	X-ray	DenseNet, SVM	96.29%
Gour Mahesh et al. [5]	X-ray	UA-ConvNet	98.02%
Rubina Sarki et al. [6]	X-ray	Vgg16, InceptionV3	93.7%, 87.5%
Mohamed Loey et al. [7]	X-ray	CNN, Bayesian Optimization	96%
Nurul Absar et al. [9]	X-ray	SqueezeNet, SVM	98.8%
Muralidharan Nehet et al. [11]	X-ray	EWT Filter, CNN	96%, 97.17%
S. V. Kogilavani et al. [13]	CT-scan	Vgg16	97.68%
Mei-Ling Huang et al. [14]	CT-scan	LightefficentNetv2	98.33%,96.33%
Hasija Sanskar et al. [16]	CT-scan	Multiclassification, CNN	98.38%
Proposed method without segmentation	X-ray	Scrateched CNN model	93.7%
Proposed Model with segmentation	X-ray, CT-scan	Scratched CNN model	98.8%,98.4%

## Data Availability

Publicly available datasets were analyzed in this study. These data can be found here: https://www.kaggle.com/datasets/tawsifurrahman/covid19-radiography-database/versions/2 (accessed on 7 July 2022).
